# High-order space-time finite element schemes for acoustic and viscodynamic wave equations with temporal decoupling

**DOI:** 10.1002/nme.4631

**Published:** 2014-02-07

**Authors:** H T Banks, Malcolm J Birch, Mark P Brewin, Stephen E Greenwald, Shuhua Hu, Zackary R Kenz, Carola Kruse, Matthias Maischak, Simon Shaw, John R Whiteman

**Affiliations:** 1 BICOM, Brunel UniversityUxbridge, UB8 3PH, England; 2 Blizard Institute, Barts and the London School of Medicine and Dentistry, Queen Mary, University of LondonEngland; 3 Clinical Physics, Barts Health National Health Service TrustEngland; 4 Center for Research in Scientific Computation, North Carolina State UniversityRaleigh, NC 27695-8212, USA; 5 Salisbury District HospitalEngland

**Keywords:** discontinuous Galerkin finite element method, spectral element method, space-time finite elements, high order methods, viscoelasticity

## Abstract

We revisit a method originally introduced by Werder *et al.* (in Comput. Methods Appl. Mech. Engrg., 190:6685–6708, 2001) for temporally discontinuous Galerkin FEMs applied to a parabolic partial differential equation. In that approach, block systems arise because of the coupling of the spatial systems through inner products of the temporal basis functions. If the spatial finite element space is of dimension *D* and polynomials of degree *r* are used in time, the block system has dimension (*r* + 1)*D* and is usually regarded as being too large when *r* > 1. Werder *et al.* found that the space-time coupling matrices are diagonalizable over 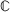 for *r ⩽*100, and this means that the time-coupled computations within a time step can actually be decoupled. By using either continuous Galerkin or spectral element methods in space, we apply this DG-in-time methodology, for the first time, to second-order wave equations including elastodynamics with and without Kelvin–Voigt and Maxwell–Zener viscoelasticity. An example set of numerical results is given to demonstrate the favourable effect on error and computational work of the moderately high-order (up to degree 7) temporal and spatio-temporal approximations, and we also touch on an application of this method to an ambitious problem related to the diagnosis of coronary artery disease. Copyright © 2014 The Authors. *International Journal for Numerical Methods in Engineering* published by John Wiley & Sons Ltd.

## 1. INTRODUCTION AND MOTIVATION

In 2001, Werder, Gerdes, Schötzau and Schwab, 1, formulated a space-time FEM for the heat equation that employed a discontinuous Galerkin (DG) method in time. This was not the first such formulation (see e.g. 2), but one particularly interesting feature of their work demonstrates that the temporal system can be diagonalised, thus making reasonably high-order finite element time discretizations feasible. Also, there are recent results 3,4 (see also the much earlier 5) showing that high-order spatial discretizations for wave equations are highly desirable in terms of the control of dispersion errors, and it is against this background that we aim here to extend the work of Werder *et al.* to facilitate high-order space-time discretizations for second-order hyperbolic wave equations. Our goal is to present a framework for useful and practical high-order space-time FEMs for a collection of common and important linear wave equation problems. We remark also that while high-order time stepping has been around for some time, by using Runge–Kutta methods for example, the approach in the succeeding text allows for the entire space-time discretization to be placed in a variational setting. This allows the arsenal of very powerful functional analytic tools relating to stability and error estimation to be deployed for the numerical simulation of wave propagation.

We deal firstly with an abstract formulation of the wave equation in Section 2 and describe the time discretization and its diagonalisation. We then apply this abstract framework to the specific examples of the acoustic wave equation and the equations of elastodynamics and viscodynamics in Section 3. The wave equation is dealt with rather swiftly in Subsection 3.1, and we move on to linear elasticity and Maxwell–Zener (hereditary) viscoelasticity in Subsection 3.2 and, finally, the decoupling procedure in the presence of both long-memory and short-memory viscoelasticity is described in Subsection 3.3.

A collection of numerical results is given and discussed in Section 4, where we aim to demonstrate the convergence rates that are achieved in the natural norms associated with the wave equation. Our presentation is entirely practical in that we do not aim to establish theorems on error bounds but rather to demonstrate the practicality of high-order finite element time stepping. Our motivation for adopting this methodology is to simulate waves in biotissue with a specific application related to the diagnosis of coronary artery disease. This application and the challenges it presents are outlined in Section 5. Section 6 then concludes with some observations. We would like to point out that this is a shorter version of the long report in 6. That report contains a much more extensive set of numerical tests.

The approach to discretization taken in the succeeding text utilises a normalized Legendre basis to effect the DGFEM in time discretization while in space we report here on the standard continuous Galerkin (CG) spatial discretization. The longer report, 6, contains results also for the case where the continuous spectral element method (SEM) using Gauss–Lobatto integration and nodes is used for space discretization.

Irrespective of the use or not of the spectral FEM, we shall see in the succeeding text (in 8 and 9) that the decoupling procedure produces a set of boundary value problems that need to be solved for each time interval. Any suitable method could be employed here, such as the standard Galerkin procedure, a ‘blended procedure’ as in 7, or indeed any of the many other tools that have been developed to ‘solve’ elliptic problems (such as, if applicable, exact solutions and asymptotics).

To provide some more context for what follows, we recall that the idea of using Galerkin finite elements in space dates back to 8 and 9 and, of course, is now a method of choice in the vast majority of cases involving elliptic operators. However, although given much less exposure, the idea of using Galerkin discretizations in time is also not new (e.g. 10–12) but it seems clear that these methods never really caught the imagination of users and producers of codes until much more recently. In fact, arguably, it was the work of Eriksson and Johnson, see 13, on adaptive space-time formulations for parabolic problems that seemed to have revived interest in these methods. The formulations in 14–18 are closely related but here we stay much closer to the crisper formulation given by Johnson in 19. We consider only DGFEM in time in this effort specifically because we are building on the work in 1, although we note that there also exist CG methods for time discretization (e.g. 20,21 and 22). These are important in their own right, due mainly to their stability and energy conservation properties, and our early results for the heat equation in 23 suggest that a ‘CGFEM-in-time’ could be developed for the wave equation. We leave this for another time.

We close this introduction by recalling one very basic reason why high-order schemes are useful. Suppose we compute up to time *T* using *N*_1_ ≫ 1 time steps. If piecewise, polynomial degree *r*_1_ is used for the approximation, then on each step we can expect the error to be of the order 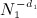 where *d*_1_ = *r*_1_ + 1. We will see in the succeeding text that *d*_1_ matrix solves are required on each time interval, and so if we (simplistically) assume that the solver time is constant in the time discretization parameters, the amount of computational work, as measured by the total number of solves, is *N*_1_*d*_1_. Now consider another set up with *N*_2_ > *N*_1_ and with polynomials of degree *r*_2_. Setting *d*_2_ = *r*_2_ + 1 and asking that the errors be ‘the same’ we have 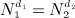 so that 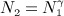 for *γ* = *d*_1_/*d*_2_ = (ln *N*_2_)/(ln *N*_1_). Because *N*_2_ > *N*_1_ ≫ 1, we must have that *γ* > 1 which, of course, means that fewer time steps are required for higher degree polynomials. The ratio of computational work needed is then 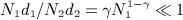 as *N*_1_ and/or *γ* become large and so we conclude that, *with sufficient solution regularity*, higher-order schemes are capable of providing higher-fidelity solutions than lower-order schemes for the same amount of computational work. We attempt to illustrate this later for the 2D examples in Section 5.

## 2. AN ABSTRACT FRAMEWORK FOR DECOUPLED DISCONTINUOUS GALERKIN FEM IN TIME

Our notation is standard and, apart from the preliminaries that follow, is introduced where necessary. Let the spatial domain of interest, Ω, be a time-independent open-bounded polytope in 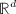 for d = 1, 2 or 3. We assume that the boundary, *∂*Ω, is partitioned into {Γ_*D*_,Γ_*N*_}(also time independent) and assume that Dirichlet boundary values are given on the closed set Γ_*D*_ with Neumann boundary values specified on the open (and possibly empty) set Γ_*N*_. As usual, we will insist that 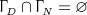 and Γ_*D*_ ∪ Γ_*N*_ = *∂*Ω. We note that, in general, there exist problems for which Γ_*D*_ may have surface measure zero, but here we insist that meas_*∂*Ω_(Γ_*D*_) > 0. Also, the unit outward normal vector to Γ_*N*_ will be written as 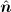. To deal with the time dependence, we set *I*: = (0,*T*] and will usually use overdots to denote partial time differentiation.

Before getting to the specific formulations of the later sections, we firstly introduce an abstract formulation along with a semidiscrete time discretization using the DGFEM. To this end, we let *V* ↪ *H* ↪ *V* ′ be a Gelfand triple of reflexive Hilbert spaces (dense and continuous embedding, see, e.g. Wloka 24) and denote the inner product and norm on *H* by 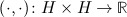 and ∥ · ∥ = (·, ·)^1/2^. We deal in this section with the standard abstract form of second-order wave equation problems in order to fix ideas and notation. Later, in Section 3, we recall some concrete applications and also some damping terms. These are important for the soft-tissue applications that we have in mind (see later in Section 5). Our goal here is to formulate a high-order finite element time discretization that is rendered practical by decoupling. However, we point out once again that such a scheme will only result in higher-solution quality if there is sufficient solution regularity.

Let 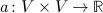 be a symmetric and *V*-coercive bilinear form and assume that, for almost every *t* ∈ *I*, we are given data *L*(*t*): *I* → *V* ′. Introducing 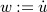 and suppressing spatial dependence for clarity, we consider the abstract problem of finding a smooth map *u*: *I* → *V* such that, 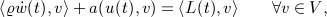
1

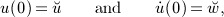
2 where 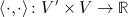 denotes the duality pairing arising from the continuous extension of (·, ·)_*H*_ to *V* ′ × *V* and 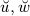 are initial data. For convenience and the avoidance of unimportant constants, we henceforth consider the space *V* as equipped with the scalar product *a*(·, ·) and the induced norm ∥ · ∥ _*V*_ = *a*(·, ·)^1/2^. In this, *ϱ* is a known positive constant that in solid dynamics represents the material's mass density. Also, and as is usual for time-dependent problems, for a Banach space 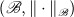, we define the 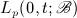 norms by 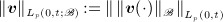. The DG-in-time formulation that follows is identical to that given by Johnson in 19. To give a little background, we recall that in 19 the time dependence of the unknown displacement and velocity is approximated by piecewise linear functions that are allowed to be *discontinuous* at the time nodes. Because each function has a first-time derivative taken, we then expect to see ‘delta functions’ at these nodes that, when integrated (as in a Galerkin formulation), produce jumps in function values. That is the reason for the jump notation that is defined and used in the succeeding text. A full and rigorous derivation of the weak formulation that follows is beyond our scope here but can be accomplished by appealing to the definition of a weak derivative or to the *Theory of Distributions* (e.g. 25).

Discretizing the time interval so that 0 = *t*_0_ < *t*_1_ < … < *t*_*N*_ = *T*, defining the time step *k*_*n*_: = *t*_*n*_ − *t*_*n* − 1_ and setting *I*_*n*_: = (*t*_*n* − 1_,*t*_*n*_) we define, as usual, the jump notation, 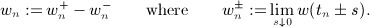


The semidiscrete DGFEM approximation of 1 is then: for each *n* = 1,2, …,*N* in turn, find 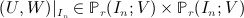 such that 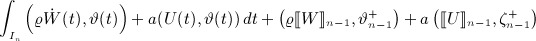


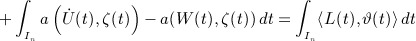


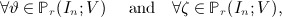
3 with the understanding that 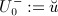 and 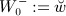. Here, for each *n*, we use 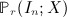 to denote the space of polynomials of degree *r* on the time interval *I*_*n*_ with coefficients in the target space *X*. The target space is omitted when 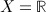, and we note that *r* could be *n*-dependent. Choosing *ϑ* = *W* and *ζ* = *U* gives, 
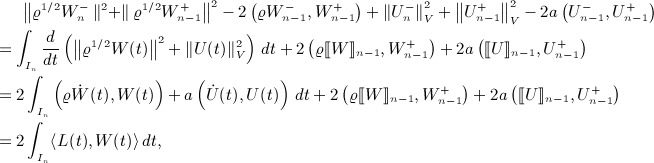
 and because 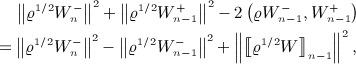 we arrive at 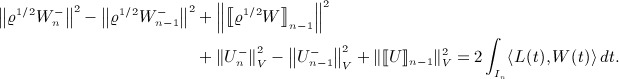 Taking *L* = 0 and summing over *n* = 1, …,*N* then gives the basic stability estimate, 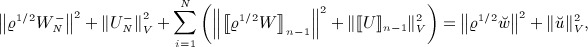 and reminds us that this scheme is dissipative. This, of course, is a general statement and holds for all polynomial degrees. It is also useful at this stage to recall Claes Johnson's result in 19 which focussed on Galerkin schemes by using discontinuous piecewise linears in time and continuous piecewise linears in space for the standard problem 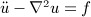 subject to homogeneous Dirichlet boundary data. Although the presentation of the *a priori* error bound, there is rather terse it seems from the comments in the succeeding text and in the introduction that we can expect the following, 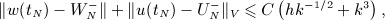
4

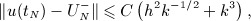
5 so long as the space-mesh is kept constant in time and where *h* is the spatial mesh size and *k* the constant time step. We will give some demonstrations of the numerically observed convergence rates later, for linear as well as higher-degree approximations, but note that we do not know of any generalization of these error bounds to higher-degree polynomials (although we might expect this to be straightforward).

We now move on to the specifics of the implementation. Let {*φ*_*i*_: *i* = 0,1, …,*r*}be a basis for 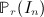 and introduce the ansatz forms of the approximations to *u* and *w* on *I*_*n*_ as, 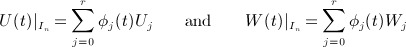 where {*U*_0_,*U*_1_, … },{*W*_0_,*W*_1_, … } ⊆ *V*. Replacing each of *ϑ*(*t*) and *ζ*(*t*) with *φ*_*i*_(*t*)*ϑ* for 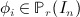 and *ϑ* ∈ *V* in 6, we obtain firstly, 
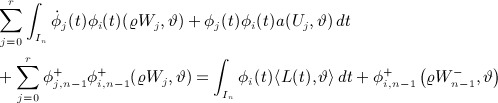
 and secondly, 
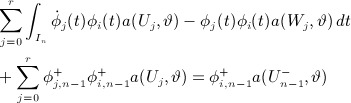
 where each holds for all *ϑ* ∈ *V* and for each *i* ∈ {0,1, …,*r*}. Define matrices *via*, 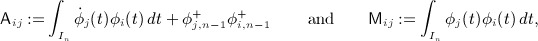 where *i* indexes the rows, and then by choosing our basis functions as the image under the linear map from [ − 1,1] to *I*_*n*_ of the normalized Legendre polynomials, we conclude easily that 2M = *k*_*n*_I. It seems useful at this point to recall the remark in 1 that these matrices are hierarchical, and also that they can be precomputed just once on a reference length and then reused for subsequent computations.

However, the main point and motivation for us here is that Werder *et al.* in 1 report that A is diagonalizable over 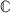 for all polynomial degrees of practical interest. This in particular means that the computational linear algebra associated with these objects is relatively cheap, and it also allows us to write 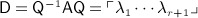 where 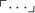 indicates a diagonal matrix of pairwise complex conjugate eigenvalues and where Q has complex entries.

So, proceeding step by step and using the abbreviation Σ_*j*_ to mean the summation 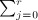, our system is 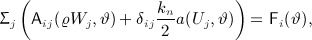
6

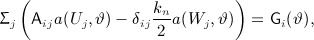
7 where 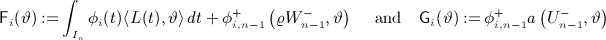 are known from data and the previous time step. Defining {*Y*_*q*_}and {*Z*_*q*_}as the unique solutions of *W*_*j*_ = Σ_*q*_Q_*jq*_*Y*_*q*_ and of *U*_*j*_ = Σ_*q*_Q_*jq*_*Z*_*q*_ we have, 
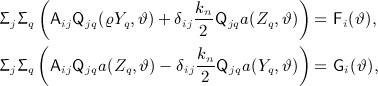
 and taking linear combinations using the rows of R: = Q^− 1^ then yields, 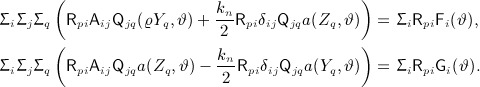 Noting that Σ_*i*_Σ_*j*_R_*pi*_A_*ij*_Q_*jq*_ = *δ*_*pq*_*λ*_*p*_ and Σ_*i*_Σ_*j*_R_*pi*_*δ*_*ij*_Q_*jq*_ = *δ*_*pq*_, setting *F*_*i*_(*ϑ*): = Σ_*p*_R_*ip*_ F _*p*_(*ϑ*) and *G*_*i*_(*ϑ*): = Σ_*p*_R_*ip*_G_*p*_(*ϑ*), we now arrive at the decoupled form 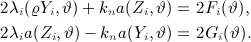 Therefore, defining 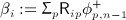, 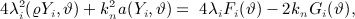
8

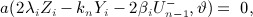
9 because 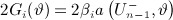.

The algorithm is now clear. At each time step, we solve *r* + 1 boundary value problems for the complex unknowns 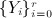 and then update with 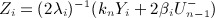. We recall that for the SEM, the mass matrices arising from the spatial discretization of these systems are diagonal, and note also that because 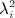 is complex we will either need to introduce complex arithmetic in the solver or treat the complex system as a two-by-two-block real system.

### Remark 2.1 non-zero Dirichlet data

Although not included in the formulation in the previous text, some of the examples in the succeeding text use non-zero Dirichlet boundary data. These data are imposed by time-projection on to the temporal basis at each desired ***x*** ∈ Γ_*D*_ to determine pointwise boundary values for each of the decoupled systems. Specifically, suppose that we want to set the known Dirichlet value 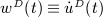 during the time interval *I*_*n*_. We assume the ansatz 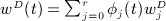 (with the superscript *D* denoting ‘Dirichlet’) and then determine the 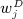 by the projection: 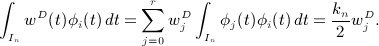


We then do the same for the 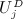 values at the boundary and we modify 17 to become, 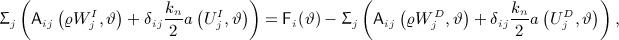
10 where 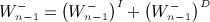 is now used in the definition of F _*i*_, and the superscript *I* denotes ‘interior’. The 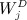 and 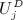 on the right are then spatially interpolated along the boundary by augmenting the spatial basis to incorporate the Dirichlet boundary nodes (with zero values in the interior), and then ‘mass’ and ‘stiffness’ (rectangular) matrix-vector contributions to the load are formed from these new inner products on the right hand side of 26. Because we derive the boundary values for *w* from 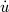, they cancel each other out in 18 and so no change is needed there. The remainder of the implementation is as described earlier but with the ‘interior’ functions being the unknowns.

#### 2.2 Remark

We could also define the semidiscrete DGFEM approximation of 1 as: for each *n* = 1,2, …,*N* in turn, find 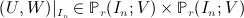 such that 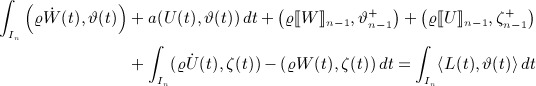
11

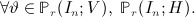
 The advantage of this is that we could deal with pure Neumann problems wherein *a*(·, ·) may no longer be coercive. Numerical tests show that this scheme works well, but we are not able to prove any stability estimates for it. For that reason, we do not consider it further here, but note that the time decoupling method described in the previous text could be applied to this formulation also.

## 3. SPECIFIC APPLICATIONS

In this section, we apply the foregoing to the specific examples of the wave equation and elastodynamics. We also enhance the formulation to take viscoelastic damping effects into account.

### 3.1 The acoustic wave equation

In this subsection, we consider the specific example problem where we seek *u* such that, 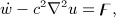


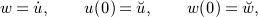


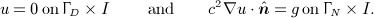


Defining *a*(*w*,*v*): = (*c*^2^ ∇ *w*, ∇ *v*), where (·, ·) is the *L*_2_(Ω) inner product, and setting 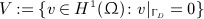 the weak form of the problem is: find *u*: *I* → *V* such that, 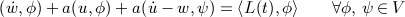 where 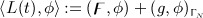. Notice that we overload (·, ·) in a completely standard way where no notational distinction is made when the arguments are scalar-valued or vector-valued.

The semidiscrete finite element formulation in 6 applies without change and so on the *n*-th time step, of width *k*_*n*_ = *t*_*n*_ − *t*_*n* − 1_ say, and for *i* = 0, …,*r*, we have to solve the boundary value problems given by: 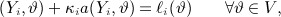 where 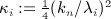, and perform the update 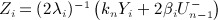. Once carried out, we obtain *W*_*i*_ = Σ_*j*_Q_*ij*_*Y*_*j*_ and *U*_*i*_ = Σ_*j*_Q_*ij*_*Z*_*j*_. In these 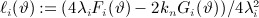, 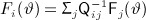 and 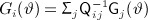 where 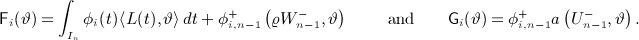


The spatial discretization has already been described in general terms earlier in Section 1, and so all that remains is to demonstrate the behaviour of the scheme. This is carried out later in Subsection 4.1 for both 1D and 2D test problems. In those experiments, we will have either *c*^2^ = *E*_0_/*ϱ* or *c*^2^ = *G*/*ϱ* where *E*_0_ and *G* represent stiffness moduli and *ϱ* a mass density (Section 5).

### 3.2 Linear elastodynamics and viscodynamics

In this section, we suppose that Ω represents the interior of a linear viscoelastic compressible body. This body is acted upon by a system of body forces 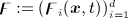 for 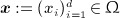 and *t* ∈ *I* and on the open (and possibly empty) set Γ_*N*_ there is prescribed a system of surface tractions 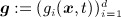 for ***x*** ∈ Γ_*N*_ and *t* ∈ *I*.

The displacement from equilibrium resulting from the action of the applied forces ***г*** and ***g*** is denoted by 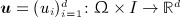 and, in this linear theory, the deformation is described by the strain tensor 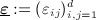 given by, 
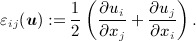
12 We assume also that *t* = 0 is a reference time such that ***u*** = ***0*** for all *t* < 0.

Newton's second law of motion with boundary conditions gives for each *i* ∈ {1, …,*d*}that, 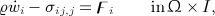
13

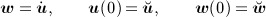


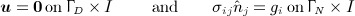
 with summation implied and where 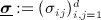 is the symmetric stress tensor.

We close this problem by recalling the standard literature on viscoelasticity (e.g. 26,27) and introducing the following linear hereditary viscoelastic constitutive relationship between stress and strain, 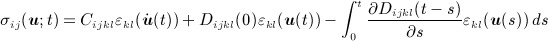
14 where, as usual, we omit the ***x*** dependence and, as in the previous text, sum over repeated indices. In this, 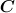 and 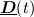 are fourth-order tensors with 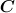 related to the Kelvin–Voigt model of viscoelasticity and 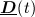 stemming from the Zener and Maxwell models.

The *Hooke's law tensor* 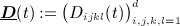 is a fourth-order stress relaxation tensor satisfying the following symmetries: 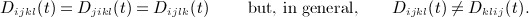
15 However, we do have *D*_*ijkl*_(*t*) = *D*_*klij*_(*t*) for *t* = 0 and *t* = ∞ in general, and for all *t* for isotropic materials (see, e.g. 28,equations (1.10), (2.62)), and the components of 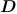 can be assumed to be (a.e. in Ω) of class 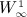 in *t* although we will not need this level of generality. In addition, because 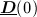 measures instantaneous linear elastic response, we follow Hooke's law and assume positive-definiteness: *γ*_*ij*_*γ*_*kl*_*D*_*ijkl*_(0) > 0 a.e. in Ω for all non-zero symmetric second-order tensors 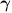.

In what follows, we will assume a much simpler version of this constitutive law. Firstly, we assume that the material is *synchronous* so that 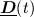 is replaced by 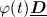, where 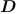 is now constant in time, and *ϕ* is a stress relaxation function. Following classical theory, we assume the Prony series form, 
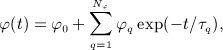
16 where 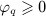 for *q* ∈ {0,1, …,*N*_*ϕ*_}, *τ*_*q*_ > 0 for *q* ∈ {1, …,*N*_*ϕ*_}, and we normalize so that 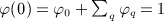. The case, *ϕ*_0_ = 0, corresponds to a viscoelastic fluid in the sense described by Golden and Graham in 26, whereas *ϕ*_0_ > 0 gives a solid. The second assumption is that the material is homogenous and so, in particular, is isotropic. This means that 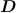 can be described by just two independent *Lamé coefficients* denoted, usually, by *λ* = *νE*/((1 + *ν*)(1 − 2*ν*)) and *μ* = 2*G* = *E*/(1 + *ν*), where *E* is Young's modulus, *G* is the shear modulus and *ν* is Poisson's ratio. Theoretically, *ν* ∈ ( − 1,1/2) but because we are dealing only with compressible non-auxetic materials, we have *ν* ∈ (0,1/2) and both *λ* and *μ* are well defined. The action of 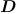 is now given specifically by *D*_*ijkl*_*ε*_*kl*_(***u***) = *λ* ∇ · ***u****δ*_*ij*_ + *με*_*ij*_(***u***), and the constitutive law can be written in the form, 
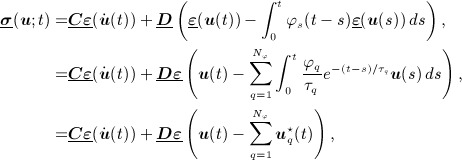
 where for *q* = 1,2, …,*N*_*ϕ*_, 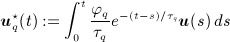 are *internal variables* (see, e.g. 29–32) and satisfy, 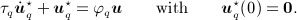
17

To give a weak formulation of this problem, we firstly define the product Hilbert spaces, ***H***^*s*^(Ω): = *H*^*s*^(Ω)^*n*^, for *s* = 0,1,2, …, with inner products given for all ***χ***, ***v*** ∈ ***H***^*s*^(Ω) by 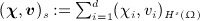. These spaces have the natural norms 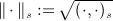 and, of course, ***L***_2_(Ω) ≡ ***H***^0^(Ω). We also use the (symmetric second-order) tensor-valued *L*_2_ space, 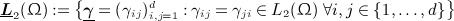 and, noting the essential boundary condition, we define the test space as, 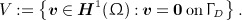
18 After integration by parts (see e.g. 33 for details), we then arrive at the weak problem of seeking a smooth map, ***u***: *I* → *V*, such that, 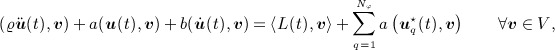
19

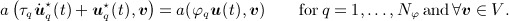
20 Here, the bilinear forms 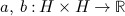 are defined by 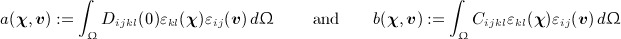 for all ***χ***, ***v*** ∈ *H*, and *L*: *I* → *V* ′ is the time-dependent linear form defined through, 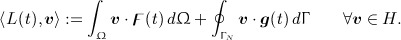


This completes the statement of the basic problem. The next section discusses the extension of the decoupling procedure to incorporate the viscoelastic terms that have just been introduced.

### 3.3 Decoupling in the presence of viscoelastic damping

Now that we have defined the viscoelastic models, we introduce them into the DG formulation as given earlier by 6 and obtain the following problem. For each *n* = 1,2, …,*N* in turn, find 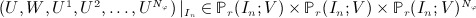 such that, 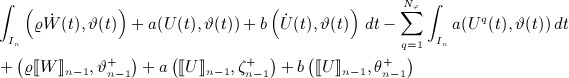
21

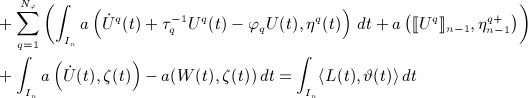


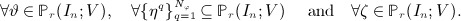
22 The decoupling is now a straightforward extension of the method described earlier and by similar means, and with the same notation for the temporal basis, we obtain firstly, 
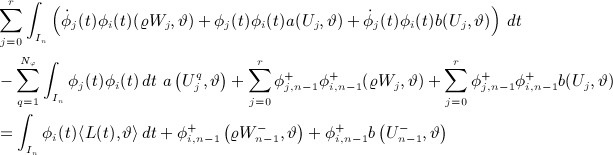
 secondly, 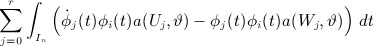


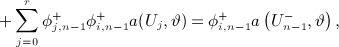
 and thirdly, 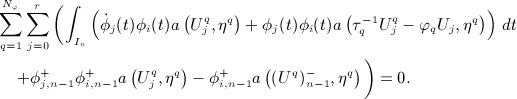


In our earlier notation, these are simply 
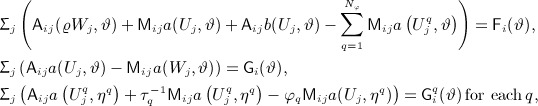
 and with easily derivable right hand side functionals F, G, 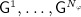. The decoupling is now carried out exactly as before.

## 4. NUMERICAL EXPERIMENTS

In this section, we give a selection of results from some computational experiments. Our main goal is to illustrate the estimated convergence rates, for the standard norms, which are achieved by this scheme. The examples have been chosen in order to try and cover all of the main possibilities. For example, we can have mixed or Dirichlet boundary data along with, in each case, various combinations of elastic and viscoelastic effects. In Subsection 4.1, we focus on the scalar-wave equation as described earlier in Subsection 3.1. Then in Subsection 4.2, we give some similar results the viscoelasticity problem with the scheme described earlier in Subsection 3.3. As for practical matters, we remark firstly that the enforcement of initial data was, in all cases, carried out by interpolation rather than projection and, secondly, that all integrals were computed by using Gauss–Legendre rules of high-enough order to render the contribution of the ‘variational crime’ negligible as compared with the errors that we are interested in. To detail this recall that the Gauss–Legendre *n* point rule is exact for polynomial integrands of degree 2*n* − 1, and we ask that this quadrature rule be exact for three orders higher than the maximum degree that occurs. This means that for an approximation based on polynomials of degree *r*, we require 2*n* − 1 = 2*r* + 3 and so *n* = *r* + 2. This is the order of rule used (in space and time — whenever necessary) in our 2D results.

As alluded to in the introduction, we have also computed results for spatial discretization using the SEM where Gauss–Lobatto rules are used to build the system matrices with nodes placed at (the tensor product of) those same Gauss–Lobatto nodes. The Galerkin method for which results are given in the succeeding text used the same node locations. This was purely for ease of implementation where the same code could perform both the Galerkin and the spectral computation.

### 4.1 The acoustic wave equation

We consider several examples, focussing first on a one-space dimensional problem in order to best reveal the (computed) temporal convergence rates. Specifically, for examples 1, 2, 3 and 4 that now follow, we consider a problem that involves different combinations of the Kelvin–Voigt and Maxwell–Zener damping. The problem, with *x*-dependence suppressed, is 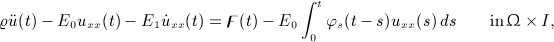
23 where Ω = (0,1) with *T* = *π* and all data chosen so that *u* = *x*(*x* − 1) cos(*t*) is the exact solution. Homogeneous Dirichlet boundary data are imposed at each end of Ω, and we use both the Galerkin and spectral spatial discretization. In these computations, we used 12 point Gauss–Legendre integration to compute the spatial inner products ‘exactly’ and, for the Galerkin spatial approximation, we used a two equal-element mesh for Ω and piecewise quadratics.

For the DG time discretization, we use piecewise polynomials of degree 1 through 7 and also compute using a Crank–Nicolson (CN), or trapezoidal, discretization of 47 and 48 to give a comparison with a standard and well-known scheme. In that, ‘CN method’, we put 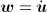 and then use central differences across a time step for the time derivatives and time nodal-averages for the remaining terms (this is a very standard discretization, see 34 for a similar approach).

We show only a small selection of the computed results here. The full set are available in the extended report 6.

#### Example 1 time error; undamped

In 59, we choose the density as *ϱ* = 1010 kg/m^3^, the stiffness modulus as *E*_0_ = 58 kPa, damping modulus as *E*_1_ = 0 Pa ⋅s and *ϕ*(*t*) = 0. To save space, the results are not shown because they are very similar to those in Example 4 in the succeeding text

#### Example 2 time error; Kelvin–Voigt

This is exactly as mentioned previously for Example 1 but with *E*_1_ = 30 kPa ⋅s. The results for the computed energy error bound given by 11 are shown in Figure 1 for the Galerkin error The SEM gave a similar picture.

**Fig 1:**
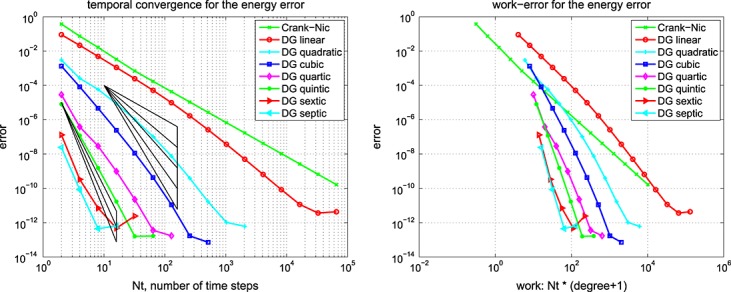
Results for the Example 2 version of 59 showing (left) the energy error, 11, and (right) the corresponding work-error dependence for the Galerkin method.

#### Example 3 time error; Kelvin–Voigt and Maxwell–Zener

Similar to Example 2 but with *ϕ*(*t*) = (1 + *e*^− *t*/0.05^)/2. The results are not shown because they are very similar to those in Example 4 in the previous text

#### Example 4 time error; Maxwell–Zener

**Fig 2:**
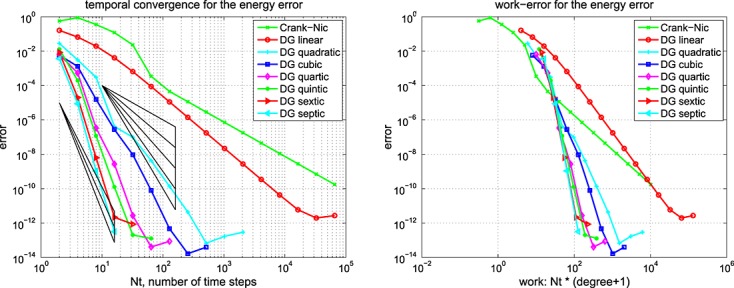
Results for the Example 2 version of 59 showing (left) the energy error, 11, and (right) the corresponding work-error dependence for the Galerkin method.

Again, similar to Example 1 but with *E*_1_ = 0, and *ϕ*(*t*) = (1 + *e*^− *t*/0.05^)/2. The results for the computed energy error bound given by 11 are shown in Figure 2 for the Galerkin error The SEM gave a similar picture.

Figures 1 and 2 show the variation of energy error with a succession of doublings of time steps (*N* = 2,4,8, … ) and also the variation of energy error with a measure of the amount of computational work (see in the succeeding text). In the convergence plots, we see two ‘fanned triangles’. These indicate the slopes that correspond to energy error bounds of size *O*(*k*^*p*^) for *p* ∈ {2,3,4,5,6}, for the fan on the right and *p* ∈ {7,8,9}, for the fan on the left.

For the work-error plots, we define work as the product of the number of time steps with the number of matrix solves required per time step. For the decoupled DG approximation using polynomials of degree *r*, this latter quantity is of course *r* + 1. For comparison purposes, we also show the performance of the CN method, recalling that it requires only one real-arithmetic solve per time step. Assuming that real-arithmetic multiplications are four times faster than complex, that real-arithmetic additions are twice as fast as complex and that banded-matrix (of dimension, *D*, say) inversion is of quadratic complexity with an equal mix of products and sums, we can estimate the operation count of a real inversion as (*D*/2)^2^ + (*D*/4)^2^ = 5*D*^2^/16 and that of a complex inversion as 2*D*^2^. The complex inversion is therefore around 32/5 = 6.4 times more expensive. In producing the work-error data for the figures, we took one complex inversion as the basic work unit (per time step) and applied the scaling of 5/32 to the CN figures to make the comparison fairer. Actual raw wall-clock timings from the Example 1 run produced a real scalings of around 1/6 to 1/6.5, so our approach appears to be well-founded. Of course, for larger and more realistic problems, an iterative solver will almost certainly be required and the work comparison becomes a much more delicate issue.

The results for the 1D problems in Examples 1, 2, 3 and 4 show the behaviour of ‘time error’ in the computed solution for four cases covering elasticity and mixtures of viscoelastic formulations. Because Examples 1 and 4 were similar, and Examples 2 and 3 were also similar, we can infer that the presence of Maxwell–Zener viscoelasticity does not alter the convergence properties of the scheme away from that displayed by the standard wave equation. The addition of a Kelvin–Voigt term, however, does seem to negatively affect the convergence behaviour but without a complete *a priori* error analysis to hand it is not clear why or how.

In particular, for Examples 1 and 4, from Figure 2, we can infer CN convergence with energy error like *k*^2^, DG(1) like *k*^3^, DG(2) like *k*^5^ and DG(3) like *k*^7^. It seems safe to conjecture a DG(*r*) energy error of order *O*(*k*^2*r* + 1^).

On the other hand, for Examples 2 and 3, from Figure 1, we can infer that the CN scheme converges with energy error like *k*^2^ and the linear DG scheme with energy error like *k*^3^. These are as expected. However, the quadratic and cubic DG schemes seem to converge like *k*^4.5^ and *k*^6^, whereas the quartic and quintic seem both to be between *k*^7^ and *k*^8^. The sextic and septic schemes appear to converge with at least *k*^9^. The reason for these unexpected behaviours is not clear although we suspect that the precision of the computations is inadequate for these high-order approximations. It does, however, seem safe to conclude that convergence is not so rapid when a Kelvin–Voigt term is present.

The accompanying work-error plots seek to illustrate the efficiency associated with higher-order methods. However, they need to be interpreted with care because it would be unlikely to have only ‘time error’ in a problem of real interest. They are included merely to remind us that the extra linear algebra workload must be borne in mind when decoupling in time. These results were for the Galerkin-in-space method. The results for the SEM were similar in both cases.

We turn now to problems in two space dimensions and an illustration of space-time error behaviour. The physical data are based on the ‘real life’ problem that we discuss later in Section 5, and the spatial domain is a scaled-up version of the one that we later describe.

For this 2D calculation, we chose the domain Ω = {0.005 < *x* < 0.15 and 0 < *y* < 0.3}with *T* = 2 and the problem 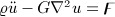 with *ϱ* = 1010 kg/m^3^ and *G* = 58 kPa. We took *N* uniform time steps and, to demonstrate the computed space-time convergence rate, we meshed the domain with an *N* by *N* grid. These numerical results use DG-in-time with piecewise polynomials in space and time of degree up to 7, with the same degree being used in both space and time except when indicated. The CN computations are based on piecewise linears for the spatial approximations, with quadratics being used in the bottom row of the figures—as explained more fully in the first example. Our motivation for mixing these degrees was to check whether there was any numerical evidence of a different convergence rate ‘in space’ than ‘in time’. The bottom rows show that in fact there is — we can expect at least one degree higher in time for the *L*_2_(Ω) error in both *u* and ∇ *u*. In the convergence graphs that follow, the triangle fans indicate convergence rates from zero to nine in half-steps.

#### Example 5 space-time error for the wave equation, Dirichlet BC's

**Fig 3:**
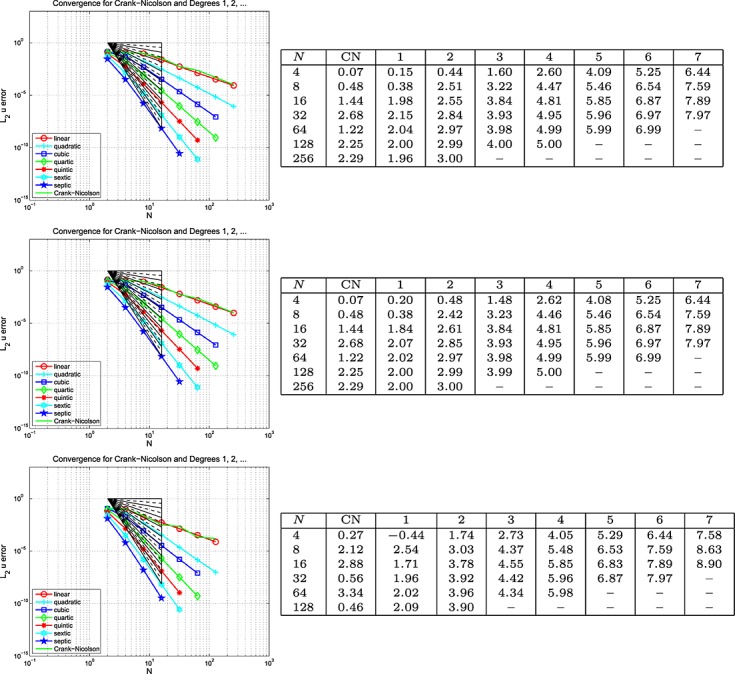
Plots of the *L*_2_(Ω) error in *u*(*T*) against *N* (equal in space and time) for the wave equation problem in Example 5 (Galerkin-in-space).

**Fig 4:**
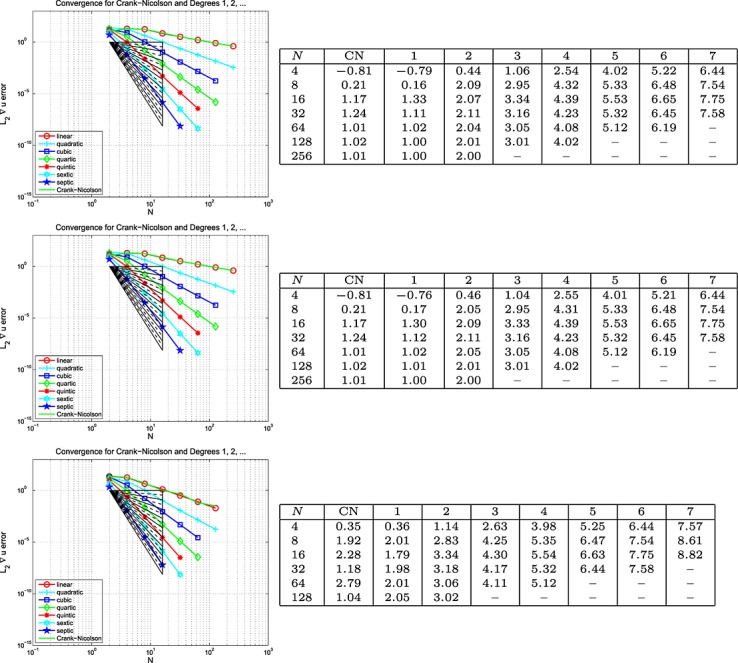
Plots of the *L*_2_(Ω) error in ∇ *u*(*T*) against *N* (equal in space and time) for the wave equation problem in Example 5 (Galerkin-in-space).

**Fig 5:**
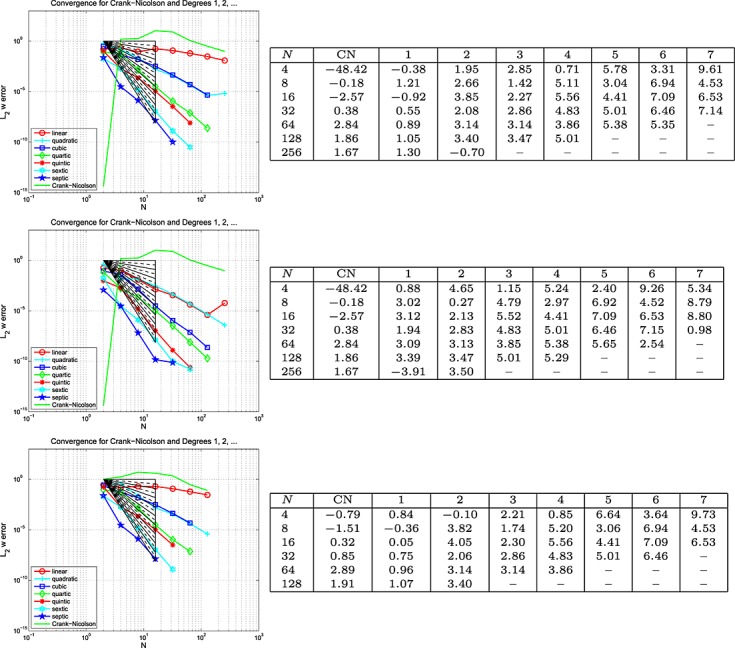
Plots of the *L*_2_(Ω) error in *w*(*T*) against *N* (equal in space and time) for the wave equation problem in Example 5 (Galerkin-in-space).

Here, Dirichlet boundary data are assumed on the whole of *∂*Ω and, *г*, the boundary and the initial data are chosen so that the exact solution is *u* = cos(2*πt*) sin(40*πx*) cos(30*πy*). The results are plotted on the left of the top row in Figures 3, 4 and 5 for the Galerkin-in-space scheme for errors in *u*(*T*), ∇ *u*(*T*) and *w*(*T*), with the estimated convergence rates tabulated on the right (Notice that a zero value of *L*_2_ error for *w* is reported for the CN scheme when *N* = 2 — this is no more than a numerical anomaly resulting from exact boundary data and a very coarse mesh.) These are based on the computed errors for *N* (first column) and for 2*N* (the results for *N* = 4 use those for *N* = 2, the row for which is not shown). The middle row of the figure shows the result of computing with one degree higher polynomials in time than in space, whereas the bottom row shows the result of using one degree higher in space than in time. The CN results are of course unaffected in the middle row. Analogous results for the spectral element scheme in the case where equal degrees are used have also been obtained and are contained in 6.

#### Example 6 space-time error for the wave equation, Mixed BC's

**Fig 6:**
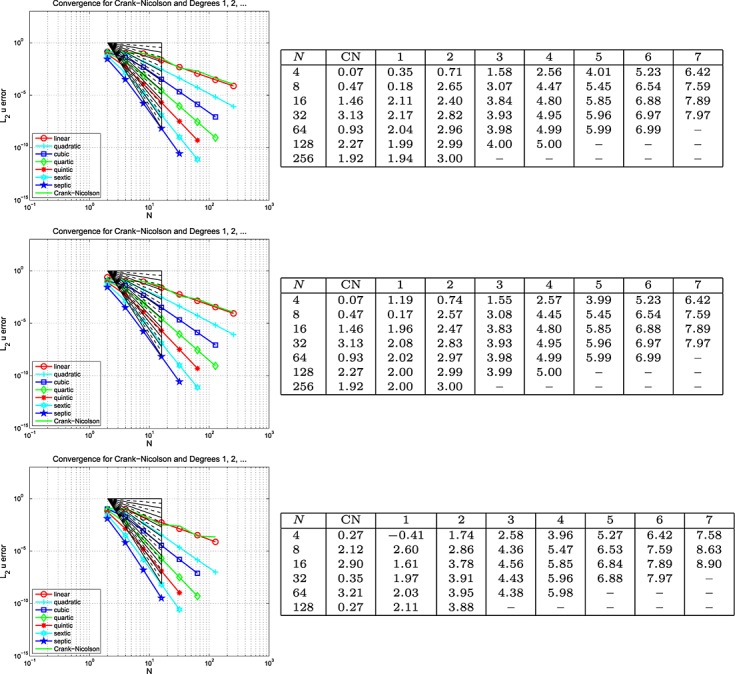
Plots of the *L*_2_(Ω) error in *u*(*T*) against *N* (equal in space and time) for the wave equation problem in Example 5 (Galerkin-in-space).

**Fig 7:**
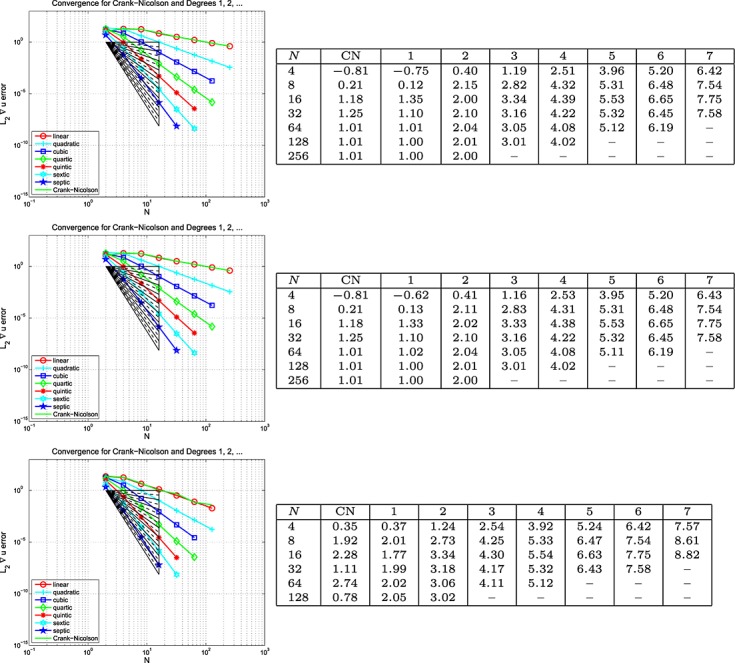
Plots of the *L*_2_(Ω) error in ∇ *u*(*T*) against *N* (equal in space and time) for the wave equation problem in Example 5 (Galerkin-in-space).

**Fig 8:**
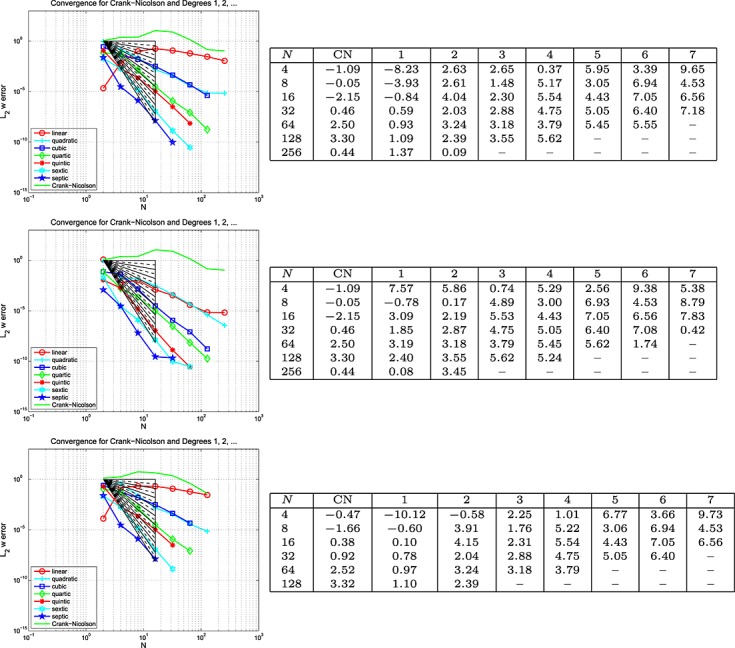
Plots of the *L*_2_(Ω) error in *w*(*T*) against *N* (equal in space and time) for the wave equation problem in Example 6 (Galerkin-in-space).

This example is set-up exactly as for Example 5, except that homogeneous (because at *x* = 0.15, we have sin(40*πx*) = 0) Dirichlet boundary data are imposed on the right-hand edge of the domain, with Neumann data everywhere else on *∂*Ω. The results are plotted on the left of the top row in Figures 6, 7 and 8 for the Galerkin-in-space scheme for errors in *u*(*T*), ∇ *u*(*T*) and *w*(*T*), with the estimated convergence rates tabulated on the right These are based on the computed errors for *N* (first column) and for 2*N* (the results for *N* = 4 use those for *N* = 2, the row for which is not shown). The middle row of the figure shows the result of computing with one degree higher polynomials in time than in space, whereas the bottom row shows the result of using one degree higher in space than in time. The CN results are of course unaffected in the middle row. Again, analogous results for the spectral element scheme are contained in 6.

With Examples 5 and 6 for the scalar wave equation, we can now discuss space-time convergence. Firstly, for Example 5, we infer from Figure 3 that 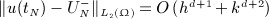 for the Galerkin-in-space scheme, with the last term being suggested by the bottom table in the figure. In a similar way, Figure 4 suggests that 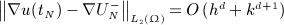, although the last term is not so clearly apparent in this case. The results for 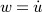 are not so useful. The curves in Figure 5 quite clearly show rapid convergence as the order increases but the tables do not give useful figures. The convergence behaviour seems too erratic to be able to draw useful conclusions. Our longer report, 6, contains the analogous results for the SEM and here we simply remark that the same conclusions can be drawn for that as for the Galerkin scheme.

Example 6 has different types of boundary data. Figures 6, 7 and 8 for the Galerkin-in-space scheme and the results for the spectral element scheme in 6 suggest essentially the same behaviour as for Example 5.

#### 4.2 Elastodynamics and viscodynamics

In this subsection, we give a numerical demonstration of this scheme for the elasticity and viscoelasticity problems described earlier in Subsection 3.2. Also, unless stated otherwise, the set-up for the numerical results is exactly as for the wave equation as described in the previous section.

##### Example 7 space-time error for viscodynamics, Mixed BC's

**Fig 9:**
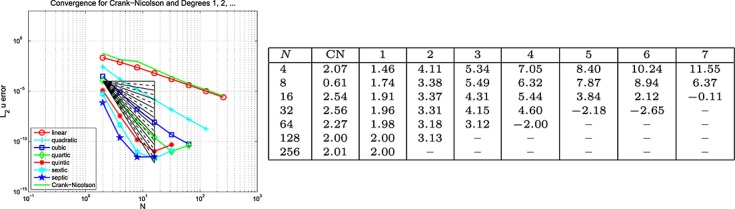
Plots of the *L*_2_(Ω) error in *u*(*T*) against *N* (equal in space and time) for the viscodynamic problem in Example 7 (Galerkin-in-space).

For this 2D calculation, we choose the same domain as previously mentioned, Ω = {0.005 < *x* < 0.15 and 0 < *y* < 0.3} but now with *T* = 0.5 and the problem given by 47 and 48. We set *ϱ* = 1010 kg/m^3^, *E* = 167 kPa, Poisson's ratio *ν* = 0.44 and include viscoelastic effects by choosing *ϕ*_0_ = 0.2, *ϕ*_1_ = 0.8 and *τ*_1_ = 0.05 in 41. The load, *г*, boundary and initial data are chosen so that the exact solution is (*u*_1_,*u*_2_)^*T*^ = (sin(2*πx*) sin(2*πy*) cos(2*πt* + *π*/4),cos(2*πx*) sin(2*πy*) sin(2*πt* − *π*/4))^*T*^ and Dirichlet data were prescribed on bottom edge of *∂*Ω with tractions prescribed elsewhere The results are given in Figures 9, 10 and 11 for the Galerkin-in-space scheme and, as before, analogous results for the SEM are in 6. These results are for the case where equal polynomial degrees in space and time were used.

**Figure:**
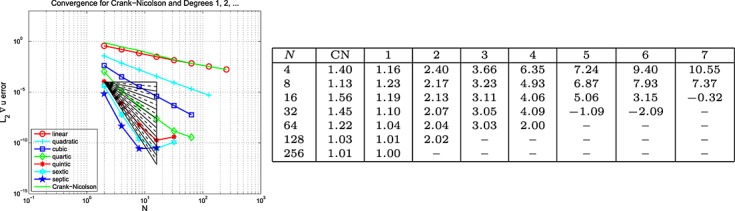
Plots of the *L*_2_(Ω) error in ∇ *u*(*T*) against *N* (equal in space and time) for the viscodynamic problem in Example 7 (Galerkin-in-space).

**Fig 1:**
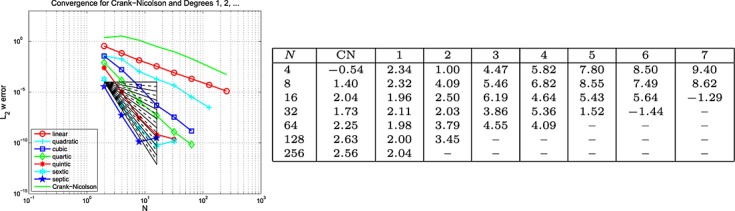
Plots of the *L*_2_(Ω) error in *w*(*T*) against *N* (equal in space and time) for the viscodynamic problem in Example 7 (Galerkin-in-space).

We observe that Figures 9, 10 and 11 for the Galerkin-in-space scheme are compatible with the expectations of error rates indicated by Examples 5 and 6. For that reason, we did not pursue a more extensive numerical study of convergence in this case (again, see 6 for the SEM).

Although these rates of convergence are, broadly, in line with what we might expect, we should sound a note of caution. A single-discretization parameter, *N*, has been used which means that the mesh width and time steps are, in essence, the same. The mixing of *h* and *k* suggested by Johnson's results (11 and 12) will therefore not be clearly revealed in our study. Also, curiously, we did not clearly observe the semi-integer rate predicted by Johnson's bounds, although it is possible that this is ‘hidden’ in the quite ragged results for *w*.

## 5. APPLICATION: SHEAR WAVES IN BIOTISSUE-MIMICKING GEL

In this section, we shift our focus away from exact errors and manufactured solutions to a much more practical experimental set-up. We consider an annular cylinder of tissue mimicking agarose gel of height 0.0514 m and with inner and outer radii 0.00175 m and 0.027 m. Here ‘height’ indicates that the cylinder is up-ended so that the radial plane is horizontal.

This experimental rig actually exists in our laboratories (see the right of Figure 2) and is the first step in an ambitious project investigating the possibility of diagnosing coronary artery disease through computational mathematics. Briefly, plaque build-up in a diseased coronary artery causes a stenosis (see the left of Figure 2) that induces a disturbance in the downstream blood flow. It is hypothesized that the resulting wall shear stresses are the cause of a 500 – 1500 Hz acoustic shear wave, or *bruit*, which travels through the thorax and which is audible on the chest wall. Our project has a long term aim to couple an inverse solver algorithm with an efficient forward solver to simulate the passage of these shear waves through viscoelastic soft tissue, produce a signal on the chest surface and, by comparing with a ‘real signal’ from a patient, use the difference in these signals to identify and locate the disease.

**Fig 12:**
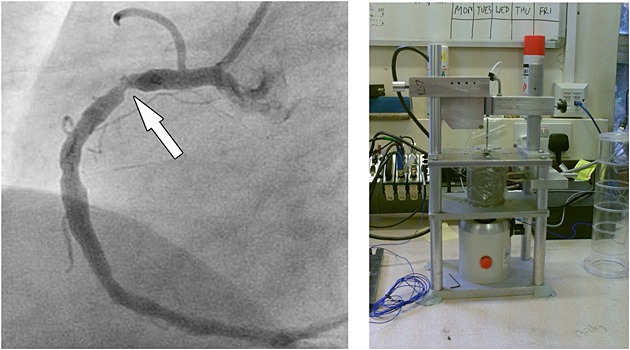
On the left (reproduced by permission from Figure 4b in 35 ©RSNA 2009), an image of a right proximal coronary artery stenosis obtained by X-Ray angiography after injecting a contrast medium through a catheter passed into the aorta and thence into the root of the coronary vessel. On the right, an illustration of our experimental rig referred to in Section 5. The middle section of the scaffold contains the gel phantom wrapped in film to prevent dehydration. The rod containing a moulded-in bead is visible emerging from the top and the unit at the bottom is the controllable ‘shaker’ that imparts the bead's vertically linear vibration. This bead is mimicked in our computational set-up by the traction in 60. The displacement of the rod and bead assembly is measured by an optical device at the top of the rig, removed in this picture for clarity.

We refer to 29,36,37 for a much more detailed treatment of the background to both the biomedical science and the proposed methodology and algorithm; to 38 and 39 for full details of the inverse problem and to 40 for the experimental protocol and findings.

The small rig referred to in the previous text, and in Figure 2, is but the first step towards this long-term objective and it is necessary to develop an efficient forward solver that can at least deal with this experimental set-up. Thus, here, we work in cylindrical polar coordinates and restrict our attention to a 2D meridian-plane cross section: we take Ω = {0.00175 < *r* < 0.027} m × {0 < *z* < 0.0514} m and we set *T* = 0.5 sec.

The bottom of the cylinder is assumed fixed in both coordinate directions (a homogeneous Dirichlet boundary condition), whereas the outer radius and top are stress free (a homogeneous Neumann boundary condition). The inner radius is subject to an oscillating traction ***g***(*t*) = (0,*g*(*t*))^*T*^ where, 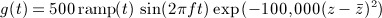
60 for 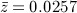 and with a frequency, *f*, as specified in the succeeding text. Here, 
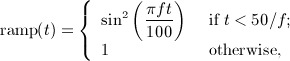
 is a ‘ramp-up’ function the role of which is to ensure that high-frequency noise associated with sudden starts does not pollute the signal — this ramp allows for the full amplitude of the traction to develop slowly over 50 full cycles. This is intended to mimic the experimental set-up.

The material data are taken as *ϱ* = 1010 kg/m^3^, *E*_0_ = 167 kPa and *ν* = 0.44 (the Voigt term was set to zero: 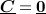). High quality estimates for the viscoelastic parameters have now appeared in 39 but, for the sake of illustration here, we simply took *ϕ*_0_ = 0.2, *ϕ*_1_ = 0.8 and *τ*_1_ = 2 with *N*_*ϕ*_ = 1 in 41. The spatial discretization used in the succeeding text is a SEM on, mostly, a 13 (radial) by 25 (axial) uniform mesh of bicubics supported on tensor products of the Gauss–Lobatto nodes. (We also ran some problems on a 25 by 50 mesh, and will return to this point later.) The DG time discretization used piecewise cubic polynomials (we refer to this as ‘DG(3)’ in the succeeding text) and all except for the spectral quadratures (the mass and stiffness matrices) use high-order (degree 12 in space and time) Gauss–Legendre rules to calculate the (space and time) definite integrals.

The imposed traction, ***g***, in 60 simulates the wall shear disturbance that may arise from a diseased (stenosed) artery, represented by the centre line of the cylinder and, in order to illustrate the performance of the DG-in-time method as developed in the previous text, we report here on the resultant displacement signals computed at the point 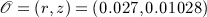 on the outer surface. This models a diagnostic sensor location on the chest surface.

For the {250,500,750,1000,1250} Hz family of frequencies, we compare the performance of the DG method against the more standard CN method. In the computations that follow, we took 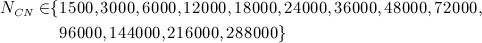 time steps for the CN method and 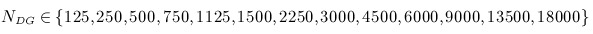 time steps for DG(3). The larger numbers were not used for the lower frequencies. The timing figures are based on using MATLAB's ®; tic and toc commands (the entire code is currently prototyped in MATLAB ®; ). Also, if we take *N* time steps up to time *T* = 0.5 to simulate a signal with a frequency, *f*, as given earlier then we have 2*N*/*f* time steps per wave. Thinking about the minimum number of data points, we need to represent such a wave we see that we must have 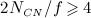, to get five data points per wave and 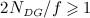 to get one cubic polynomial per wave. It follows that the minimum number of time steps for each given frequency is then, 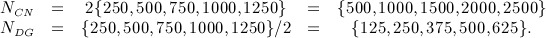


Selections from the computed signals are shown for *f* = 250 Hz and a 25 × 50 mesh in Figure 3, and for a 13 × 25 mesh in Figure 4. For *f* = 1250 Hz, selected output is shown in Figure 5, for the 13 × 25 mesh, whereas for the 25 × 50 mesh, we show output in Figure 6. Results for *f* ∈ {250,500,750} Hz are given in 6.

**Fig 13:**
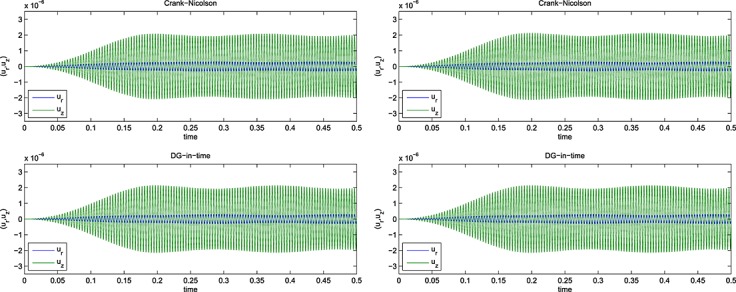
Computed signals for 250 Hz on a 25 × 50 mesh with (left) 6000 CN and 500 DG(3) and (right) 12000 CN and 750 DG(3) time steps.

**Fig 14:**
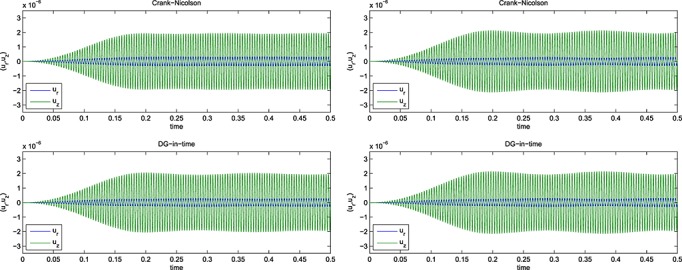
Computed signals for 250 Hz on a 13 × 25 mesh with (left) 6000 CN and 500 DG(3) and (right) 12000 CN and 750 DG(3) time steps.

**Fig 15:**
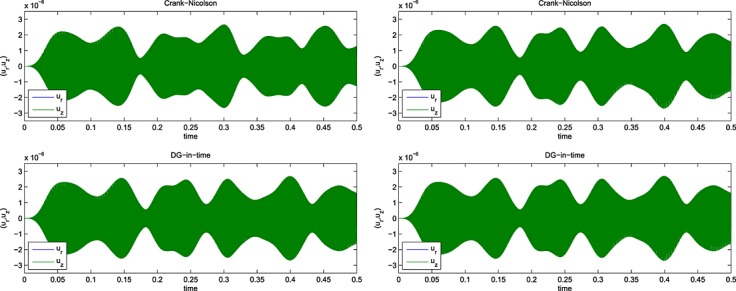
Computed signals for 1250 Hz on a 13 × 25 mesh with (left) 48000 CN and 3000 DG(3) and (right) 288000 CN and 18000 DG(3) time steps.

**Fig 16:**
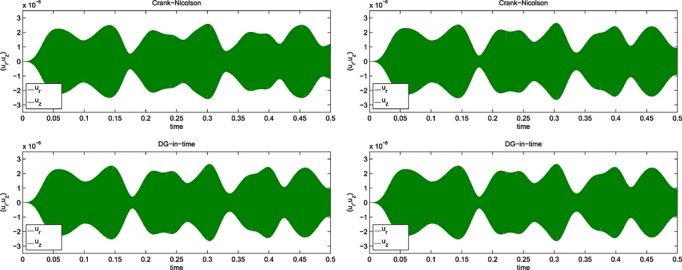
Computed signals for 1250 Hz on a 25 × 50 mesh with (left) 72000 CN and 4500 DG(3) and (right) 288000 CN and 18000 DG(3) time steps.

To discuss these results, we begin with the computations for the least challenging frequency, 250 Hz, where we find output results for a selection of time step sizes and for the 25 × 50 spatial mesh in Figure 3. The corresponding results for the 13 × 25 mesh are shown in Figure 4.

In a crude ‘eyeball judgement’, we can see that the DG(3) scheme seems to have converged to a practically useful level of accuracy, and with both meshes, at 500 time steps, whereas CN requires around 12000 for both meshes. This equates to 2000 complex system solves for DG(3) as against 12000 real solves for CN.

We can use this observation to infer that it is reasonable to assume that the DG solution for the largest *N*_*DG*_ is much more accurate than the CN solution at all considered values of *N*_*CN*_. This suggests a method for approximating the error in these computed signals. For each of the frequencies, we take the DG solution corresponding to the largest *N*_*DG*_ in use, call these 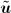 and *N*_*DG*,max_, and assume that it is ‘exact’. We then approximate the error in the CN and DG solutions on the set, 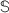, of discrete times that are common to the ‘exact’ computation and the one being assessed. With these notions, we define the relative radial (subscript *j* = 1) and axial (subscript *j* = 2) errors by, 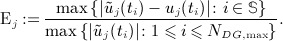


The results for the resulting approximate relative error max{E_1_,E_2_}are shown in Figures 7 and 8 for the 24 × 50 mesh and the 13 × 25 mesh. We can see that they are effectively identical and, of course, we caution against paying too much attention to the estimated relative errors associated with the larger values of *N*_*DG*_. We also recorded the run times using MATLAB's ®; tic and toc wall-clock timer commands and although this allows the execution times to be plotted against number of time steps, we consider it more interesting to plot error as a function of execution time.

**Fig 17:**
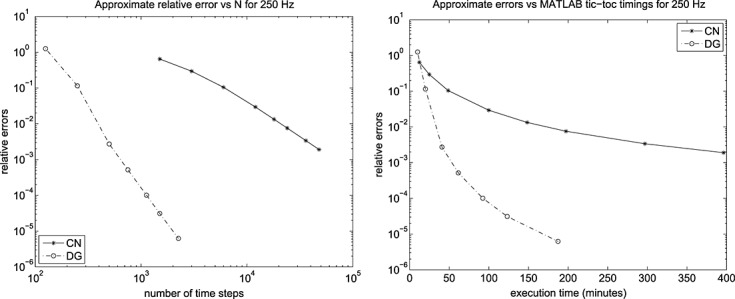
Approximated errors and ‘tic-toc’ MATLAB ®; wall-clock timings in minutes for the 250 Hz run with CN and DG(3) on a 25 × 50 mesh.

**Fig 18:**
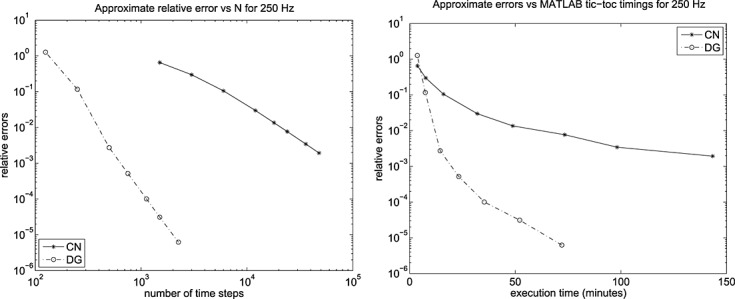
Approximated errors and ‘tic-toc’ MATLAB ®; wall-clock timings in minutes for the 250 Hz run with CN and DG(3) on a 13 × 25 mesh.

Here, for the 25 × 50 computation, we see from the left of Figure 7 that to obtain a relative error of about 10^− 3^ we need around 500 DG(3) steps and 48000 CN steps. The right hand plot tells us that, for these values, CN took about 400 min: about 10 times that of DG(3).

Because Figure 7 and Figure 8 are essentially the same (except, of course, for execution time), we can assume that we have in effect computed an ‘exact solution’ that gives us confidence in our findings at 250 Hz.

In 6, we give 13 × 25 mesh results for 500 Hz, 750 Hz and 1000 Hz but include only the results for the highest frequency we computed for, 1250 Hz, here. In that case, the error and timing data are shown in Figure 9 for the 13 × 25 mesh and in Figure 10 for the 25 × 50 mesh.

**Fig 19:**
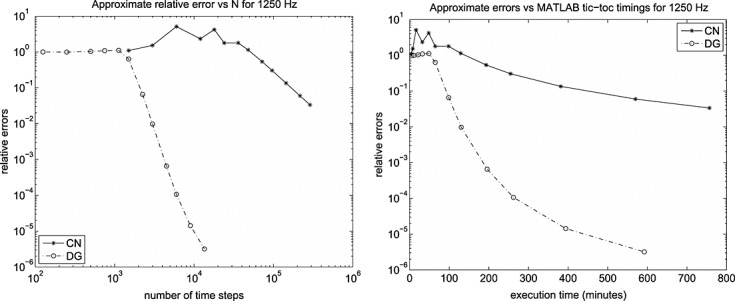
Approximated errors and ‘tic-toc’ MATLAB ®; wall-clock timings in minutes for the 1250 Hz run with CN and DG(3) on a 13 × 25 mesh.

**Figure:**
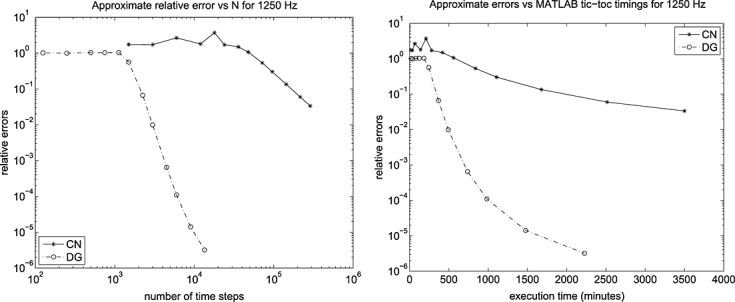
Approximated errors and ‘tic-toc’ MATLAB ®; wall-clock timings in minutes for the 1250 Hz run with CN and DG(3) on a 25 × 50 mesh.

It seems clear that on the 13 × 25 mesh the DG(3) results have converged by 3000 time steps and CN by 288 000; whereas for the 25 × 50 mesh, we would estimate these figures at 4500 and 288 000. However, the ‘converged plots’ are not the same — there are significant differences in the wave envelopes for times later than 0.2. This is a clear indication that a finer spatial mesh is needed in order to properly assess the situation at higher frequencies. This study is ongoing but will have to wait for a software development phase to be completed: the execution times required by MATLAB ®; for repeating all these tests on a, say, 50 × 100 mesh, are prohibitive.

## 6. CONCLUSIONS AND DISCUSSION

The conclusions relating to the specific numerical performance of the scheme have been included in the previous two sections. Here, to close, we would like to make just two more general remarks.

Firstly, despite the need to introduce complex arithmetic, it seems clear that DG(3) and, by extension, DG(*p*), can offer much greater efficiency for certain types of wave equation problem. Linearity, solution smoothness and (temporally) constant coefficients are the obvious desirable properties, and we note that in engineering dynamics these are often satisfied. The extension and study of the technique beyond these assumptions seems worthwhile, as does a full and detailed *a priori* error analysis building on that in 19. On the basis of the results in the previous text, we could conjecture a temporal convergence rate at the nodes corresponding to a *O*(*k*^2*q* + 1^) error term (as for the DG scheme for parabolic problems in 13) when the Kelvin–Voigt term is not present. It also seems clear that the addition of Maxwell–Zener viscoelastic damping does not affect the convergence rates.

Secondly, and to close, we note that the scheme developed in the previous text is suited to both coarse and fine grained parallelism in the sense that each of the decoupled problems can be solved in parallel (the fine graining). Because these problems are independent of each other, they can be solved simultaneously on separate hardware (the coarse graining).
